# The Mechanism of Chromatin Remodeler SMARCAD1/Fun30 in Response to DNA Damage

**DOI:** 10.3389/fcell.2020.560098

**Published:** 2020-09-25

**Authors:** Ze-Bin Tong, Hua-Song Ai, Jia-Bin Li

**Affiliations:** ^1^College of Pharmaceutical Sciences, Soochow University, Suzhou, China; ^2^Ministry of Education Key Laboratory of Bioorganic Phosphorus Chemistry and Chemical Biology, Department of Chemistry, Tsinghua University, Beijing, China

**Keywords:** SMARCAD1/Fun30, chromatin remodeler, DNA damage, DNA end-resection, single-stranded DNA, post-translational modification

## Abstract

DNA packs into highly condensed chromatin to organize the genome in eukaryotes but occludes many regulatory DNA elements. Access to DNA within nucleosomes is therefore required for a variety of biological processes in cells including transcription, replication, and DNA repair. To cope with this problem, cells employ a set of specialized ATP-dependent chromatin-remodeling protein complexes to enable dynamic access to packaged DNA. In the present review, we summarize the recent advances in the functional and mechanistic studies on a particular chromatin remodeler SMARCAD1^Fun30^ which has been demonstrated to play a key role in distinct cellular processes and gained much attention in recent years. Focus is given to how SMARCAD1^Fun30^ regulates various cellular processes through its chromatin remodeling activity, and especially the regulatory role of SMARCAD1^Fun30^ in gene expression control, maintenance and establishment of heterochromatin, and DNA damage repair. Moreover, we review the studies on the molecular mechanism of SMARCAD1^Fun30^ that promotes the DNA end-resection on double-strand break ends, including the mechanisms of recruitment, activity regulation and chromatin remodeling.

## Introduction

In eukaryotes, DNA is highly packaged to form chromatin. Nucleosomes are the basic units of chromatin, consisting of roughly 1.75 super-helical turns of DNA wrapped around octamers composed of four core histones: one histone H3-H4 tetramer and two histone H2A-H2B dimers (Kornberg, 1974; Kornberg and Thomas, 1974). Biological processes such as DNA replication, transcription, and DNA damage repair all occur at the chromatin level. However, the highly compacted state of chromatin hinders the access of corresponding regulatory factors to DNA. Chromatin remodelers utilize the energy of ATP to change the structure of chromatin, thereby ensuring the normal progress of nuclear processes (Clapier and Cairns, 2009; Hargreaves and Crabtree, 2011).

Chromatin remodelers that have a conserved ATPase domain belong to the DNA helicase superfamily 2. Phylogenetic and functional analyses have divided the remodelers into four subfamilies: imitation switch (ISWI), chromo-domain helicase DNA-binding (CHD), switch/sucrose non-fermentable (SWI/SNF) and INO80. Each subfamily uses different mechanisms to achieve nucleosome positioning, sliding, ejection, or changes in nucleosome composition (Clapier and Cairns, 2009; Hargreaves and Crabtree, 2011; Bartholomew, 2014; Clapier et al., 2017). For instance, remodelers of the INO80 subfamily remove a particular histone within a nucleosome and replace it with either a canonical or a variant histone. SWI/SNF subfamily remodelers primarily render the chromatin more accessible to proteins through sliding nucleosomes along with the DNA, evicting nucleosome components (such as H2A-H2B dimers) or ejecting full nucleosomes (Clapier et al., 2017). Recently, the structural and mechanistic studies of chromatin remodelers have gained much attention. Up to now, the structures of chromatin remodelers in almost all subfamilies in yeast and humans have been determined, such as the RSC complex of the SWI/SNF subfamily, INO80 complex and SWR1 complex of the INO80 subfamily, and Chd1 of the CHD subfamily (Farnung et al., 2017; Liu et al., 2017; Ayala et al., 2018; Eustermann et al., 2018; Feng et al., 2018; Willhoft et al., 2018; Li et al., 2019; Yan et al., 2019; Ye et al., 2019; He et al., 2020), which brings new insights into how the chromatin remodelers bind to the nucleosomes, and how they translocate DNA to break histone-DNA contact for specific outcomes (slide, exchange, position) (Bartholomew, 2014; Zhou C.Y. et al., 2016). Nonetheless, it remains to elucidate how the activity of remodelers is regulated by chromatin factors or post-translational modifications.

Fun30 is a newly discovered chromatin remodeler (Awad et al., 2010) and has been proved to play an important role in a variety of cellular processes (Rowbotham et al., 2011; Chen X. et al., 2012; Costelloe et al., 2012; Lee et al., 2017). Initially, *fun30* was a gene discovered during the whole genome sequencing of budding yeast. Fun30 is highly conserved in different species, including yeast, arabidopsis, nematodes, zebrafish, mouse, and human. The human homolog, SMARCAD1 (SWI/SNF-related, Matrix-associated, Actin-dependent Regulator Chromatin group A, contain DEAE box) has been mapped to the chromosome 4q22-q23 region, which is rich in breakpoints and deletion mutants of genes involved in several human diseases (Adra et al., 2000). SMARCAD1 mutations or deletions are closely related to many diseases, such as breast cancer and some autosomal-dominant inherited diseases, e.g., Adermatoglyphia and Basan Syndrome (Nousbeck et al., 2011; Marks et al., 2014; Al Kubaisy et al., 2016; Arafat et al., 2018).

Moreover, SMARCAD1 is a vital regulator in many cellular processes including DNA replication, transcription regulation, establishment and maintenance of heterochromatin, and DNA damage repair (Rowbotham et al., 2011; Chen X. et al., 2012; Costelloe et al., 2012; Doiguchi et al., 2016). This raises many interesting questions regarding the molecular mechanism by which SMARCAD1 regulates various cellular processes and how its ATPase motor domain is regulated. Comprehension of the detailed molecular mechanisms of SMARCAD1^Fun30^-dependent regulation is believed to be of importance for fundamental research and disease treatment.

In this review, we summarize the recent studies on how SMARCAD1^Fun30^ regulates various cellular processes through its chromatin remodeling activity, especially the regulatory role of SMARCAD1^Fun30^ in DNA damage repair.

## The Domain Architectures and Biological Functions of SMARCAD1^Fun30^

**FIGURE 1:**
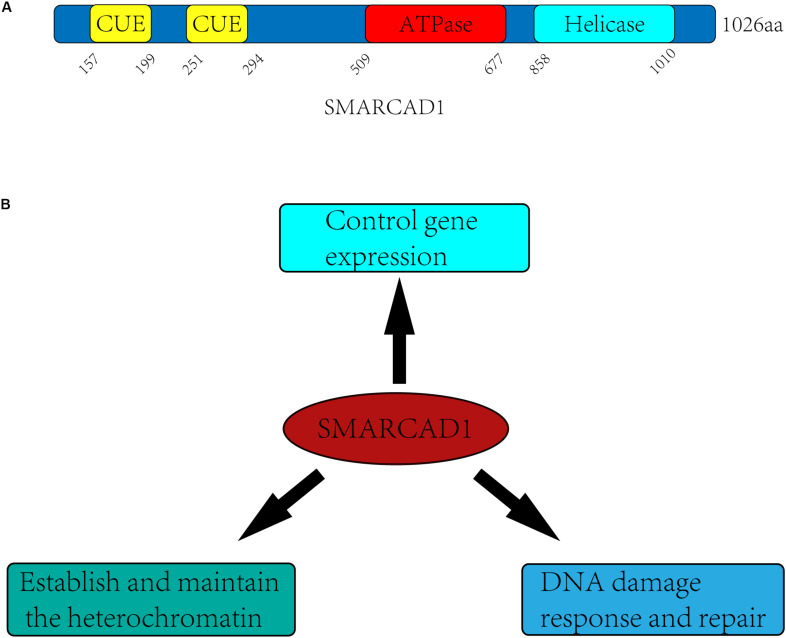
Domain architectures and biological functions of the human SMARCAD1. **(A)** SMARCAD1 contains two CUE domains (aa 157-199, aa 251-294), which probability engage in ubiquitinated protein interactions. The ATPase domain (aa 509-677) and helicase domain (aa 858-1010) are catalytic domain, and there is an insert (aa 677-858) between the ATPase domain and the helicase domain. **(B)** The biological functions of SMARCAD1 mainly include controlling gene expression, establishing and maintaining the heterochromatin, and promoting DNA damage repair.

SMARCAD1 and its orthologs contain approximately 1000 amino acids, which have a highly conserved ATPase domain (aa 509-677) and a helicase domain (aa 858-1010) (Figure 1A). In yeast, Fun30 is a single-subunit chromatin remodeler and acts in a homodimeric form (Awad et al., 2010). However, in humans, no *in vitro* experiments have demonstrated that SMARCAD1 alone has chromatin remodeling activity. When SMARCAD1 was purified from HEK293 cells, some subunits of other chromatin remodeler complexes were found, such as RUVBL1, RUVBL2, and ACTL6A (Rowbotham et al., 2011). Indeed, most chromatin remodelers work in multi-subunit complexes, which raises the possibility that SMARCAD1 may function in the form of complexes and its recruitment may be affected by these subunits. Bioinformatics analysis showed that the ATPase domain and helicase domain of yeast Fun30 share a high degree of homology with Swr1 and Ino80 (Flaus et al., 2006). Moreover, SMARCAD1 and almost all its orthologs are predicted to contain ubiquitin-binding CUE (Coupling of Ubiquitin conjugation to Endoplasmic reticulum degradation) domains, which probably engage in interaction with ubiquitinated proteins (Neves-Costa et al., 2009).

It has been confirmed in previous studies that SMARCAD1 and its orthologs mainly function by altering the structure of chromatin (Awad et al., 2010; Byeon et al., 2013; Steglich et al., 2015; Lee et al., 2017). They can not only regulate gene expression by promoting dissociation of nucleosomes, but also make an important contribution to gene silencing of centromeres, telomeres, and other regions (Neves-Costa et al., 2009; Rowbotham et al., 2011; Yu et al., 2011; Durand-Dubief et al., 2012; Steglich et al., 2015; Lee et al., 2017; Ding et al., 2018; Jahn et al., 2018). SMARCAD1 and its orthologs mediated gene silencing is mainly achieved by maintaining heterochromatin markers on nucleosomes (Durand-Dubief et al., 2012; Taneja et al., 2017; Sachs et al., 2019) or recruiting the methyltransferase G9a to modify newly synthesized histones (such as H3K9me3) for establishing heterochromatin *de novo* during DNA replication (Rowbotham et al., 2011; Yu et al., 2011). There is evidence that SMARCAD1 can also promote the formation of euchromatin, indicating the dual role of SMARCAD1 in the regulation of chromatin structure (Xiao et al., 2017). In addition, SMARCAD1 and its yeast ortholog Fun30 play a central role in promoting long-range resection of DNA (Chen X. et al., 2012; Costelloe et al., 2012; Eapen et al., 2012). This process may require the eviction of nucleosomes and the destruction of chromatin to facilitate the production of single-stranded DNA by nucleases, which is the initial step in DNA damage repair by homologous recombination (Sun et al., 2020). The diverse functions of SMARCAD1 are likely to depend on different remodeling mechanisms (sliding, positioning, dimer exchange) (Figure 1B).

### SMARCAD1^Fun30^ Controls Gene Expression

In 2017, a complex that acetylates histone H2A in an ATP-dependent manner was purified from *Drosophila* nuclear extracts and found to contain SMARCAD1 and histone acetylases P300/CBP. *In vitro* experiments showed that SMARCAD1 enhanced CBP-catalyzed H2A K5 and K8 acetylation in an ATP-dependent manner to promote transcription. Also *in vivo*, Chip-seq and *Drosophila* genetic experiments revealed that SMARCAD1 and CBP are co-localized, and interact with each other (Doiguchi et al., 2016). Therefore, SMARCAD1 is inferred as a transcriptional activator.

Through genetic screening, Lee et al. found that Fft3 (SMARCAD1 homolog in fission yeast) regulates RNAPII occupation in the transcription region. The discriminate of nucleosome occupancy between WT and *fft3*Δ cells further revealed that Fft3 promotes RNAPII transcription mainly by hydrolyzing ATP to disrupt histone-DNA interactions to induce nucleosome disassembly (Lee et al., 2017).

Although some roles of SMARCAD1 and its orthologs in regulating gene expression have been mentioned, the overall importance of their contribution to transcription regulation remains to be elucidated. At least in budding yeast, the absence of Fun30 caused only minor changes in the expression of few proteins (Chen X. et al., 2012), possibly reflecting redundancy effects with other nucleosome remodelers (Barbaric et al., 2007; Smolle et al., 2012). Therefore, the function of SMARCAD1 and its orthologs in promoting transcription needs to be further studied.

### SMARCAD1^Fun30^ Establishes and Maintains the Heterochromatin

Different from the role of SMARCAD1 in transcription activation, it is surprising and interesting that SMARCAD1 and all of its orthologs can localize heterochromatin and contribute to the establishment and maintenance of heterochromatin (Neves-Costa et al., 2009; Rowbotham et al., 2011; Stralfors et al., 2011; Yu et al., 2011; Durand-Dubief et al., 2012; Steglich et al., 2015; Taneja et al., 2017; Ding et al., 2018; Jahn et al., 2018; Sachs et al., 2019).

Rowbotham et al. (2011) used FLAG-affinity protein purification in HEK293 cells to identify the interaction partners of SMARCAD1. Mass spectrometry revealed that the SMARCAD1 interacting proteins mainly include heterochromatin maintenance factor KAP1, deacetylase HDAC1/2, and H3K9 methylase G9a. Interestingly, knockout of SMARCAD1 increased the acetylation level of H3/H4 but reduced the methylation level of H3K9, which was considering as a mark of transition from euchromatin to heterochromatin (Filion et al., 2010; Fierz, 2014). These results suggested that SMARCAD1 may cooperate with HDAC1/2 and G9a to regulate the level of histone modifications, therefore inducing the formation of heterochromatin. Moreover, it was reported that SMARCAD1 was recruited to the sites of DNA replication by directly interacting with PCNA (Rowbotham et al., 2011), a central component of the replication machinery. During the DNA replication process, SMARCAD1 recruits the G9a/GLP to methylate the newly synthesized histones, thus establishing the H3K9 methylation and heterochromatin *de novo*. Overall, these results indicated the important role of SMARCAD1 in the formation of heterochromatin.

Notably, SMARCAD1 and its orthologs are not only involved in *de novo* generating heterochromatin but also participate in the maintenance of heterochromatin or silent chromatin (Neves-Costa et al., 2009; Stralfors et al., 2011; Durand-Dubief et al., 2012; Taneja et al., 2017). For example, Fun30 and Fft3 are essential for maintaining a proper chromatin structure at centromeres and subtelomeres, rDNA repeats, and mating type loci (Neves-Costa et al., 2009; Stralfors et al., 2011; Durand-Dubief et al., 2012; Steglich et al., 2015; Taneja et al., 2017; Jahn et al., 2018). In 2017, Fft3 was identified as a factor to affect heterochromatin maintenance through genetic screening. It was further demonstrated by nucleosome exchange assay that Fft3 mainly inhibits the parent histones dilution in the progeny DNA during DNA replication, thereby promotes the inheritance of the heterochromatin marker (Taneja et al., 2017). When Fft3 is defective, euchromatin invades the centromere and subtelomeres, causing the change of histone modifications, incorrect incorporation of histone variants, misregulation of gene expression, and severe chromosome segregation defects (Stralfors et al., 2011). Moreover, Fun30 has also been found to ensure the formation of the correct centromere structure by inhibiting the transcription of genes in the centromere region and to ensure the correct separation of chromosomes (Neves-Costa et al., 2009; Durand-Dubief et al., 2012).

### SMARCAD1^Fun30^ Is Involved in DNA Damage Response and Repair

In addition to the functions mentioned above, the role of SMARCAD1 in DNA damage repair has also attracted attention and been more thoroughly studied. In 1999, Fun30 was identified in budding yeast by genetic screening to affect chromosomal stability and segregation (Ouspenski et al., 1999). This is the first connection of Fun30 to the DNA damage response. Fun30 is not essential for the survival of yeast, but its loss can lead to genomic instability (Stralfors et al., 2011; Durand-Dubief et al., 2012). Interestingly, overexpression of Fun30 was also shown to lead to genomic instability (Ouspenski et al., 1999). Besides, SMARCAD1 overexpression has been observed in a retroviral E1A-expressing HeLa cell line with an increased capacity for gene reactivation by genomic rearrangements, suggesting a role for SMARCAD1 in genomic instability.

The core of DNA damage signaling is a pair of related protein kinases, ATM (ataxia telangiectasia mutated) and ATR (ATM and Rad3-related), which are activated by DNA damage. Matsuoka et al. (2007) screened the potential substrates of ATR and ATM through peptide IP and SILAC technology and identified SMARCAD1 involving in DNA damage repair. Fun30 and SMARCAD1 were further connected to the DNA mismatch repair pathway (MMR) and shown to be required for the resistance to irradiation and camptothecin (CPT) (Neves-Costa et al., 2009; Costelloe et al., 2012; Chen Z. et al., 2016; Chakraborty et al., 2018; Terui et al., 2018). These results indicated that SMARCAD1^Fun30^ may be essential for DNA damage repair. This poses an interesting question as to how SMARCAD1 promotes DNA damage repair.

In 2012, a series of pioneering studies established a key role of Fun30 and SMARCAD1 in DNA damage repair (Chen X. et al., 2012; Costelloe et al., 2012; Eapen et al., 2012). Fun30 was discovered by performing a yeast genomic screen using a plasmid-based assay to identify novel proteins that are important in homologous recombination. Knock out of Fun30 caused defects in the DNA end-resection, a key step of homologous recombination. As an ATP-dependent chromatin remodeler, SMARCAD1^Fun30^ was speculated to facilitate the role of DNA end-resection factors by altering the structure of chromatin, thereby promoting the process of DNA end-resection. This speculation has indeed been confirmed by *in vivo* experiments. SMARCAD1^Fun30^ and its ATPase activity are both essential for efficient long-range resection (Chen X. et al., 2012; Costelloe et al., 2012; Eapen et al., 2012; Bantele et al., 2017).

Notably, Chen et al. found that one of the main functions of Fun30 is to antagonize the DNA end resection inhibitor Rad9, a member of the DNA damage checkpoint. The DNA damage checkpoint is a signal transduction pathway that communicates the occurrence and severity of DNA damage to the cell and activates the subsequent cellular response. This cascade is initiated by the sensor proteins Ku/MRX, RPA and 9-1-1 complex, which are recruit to DNA damage sites by specifically recognizing the damage-specific DNA structures. For example, Ku/MRX is recruited by specifically recognizing the ends of DNA double-strand breaks, RPA is recruited by ssDNA, and 9-1-1 is loaded at the ss-dsDNA junction. Subsequently, these sensor proteins recruit the apical kinases Mec1^ATR^ and Tel1^ATM^. Moreover, the mediator proteins Rad9 and scaffold protein Dpb11 are also recruited to DNA damage sites (Bantele, 2018).

**FIGURE 2:**
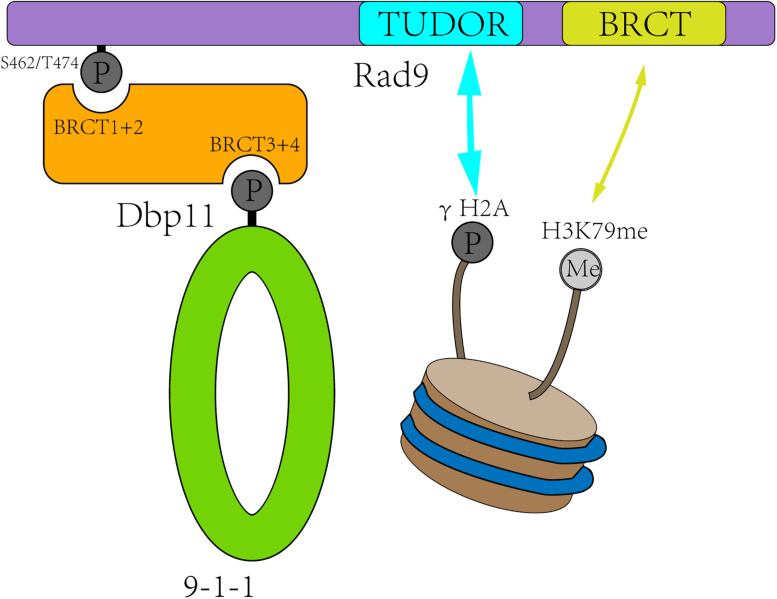
Rad9 is recruited to the DSB sites in two ways. First, CDK-dependent Dbp11 phosphorylation on S462/T474 residues promote Rad9 binding to the BRCT1+2 domain of Dbp11 to form a ternary checkpoint complex (green and orange). Second, Rad9 recognized modified histones in a bivalent mode, with its TUDOR domain (blue) recognizing H3K79me and its C-terminal BRCT domain (yellow) interacting with γH2A.

Rad9 (structurally and functionally equivalent to human MDC1/BRCA1/53BP1) is the first identified checkpoint player (Weinert and Hartwell, 1988). It is associated with damaged chromatin in two ways (Figure 2). First, Rad9 is recruited to the DSB sites by interacting with Tel1-mediated H2A S129 phosphorylation and Dot1-catalyzed H3K79 mono-methylation (H3K79me) (Wysocki et al., 2005; Toh et al., 2006; Grenon et al., 2007; Hammet et al., 2007). Second, in S, G2, and M phases, Rad9 is recruited to DSB sites through direct interaction with the S462 and T474 phosphorylated BRCT1+2 domain of Dbp11 in a CDK-dependent manner (Granata et al., 2010; Pfander and Diffley, 2011).

Then, the damaged chromatin-anchored Rad9 provides a recruitment platform for DSB response proteins and activates the subsequent checkpoint signals. For example, phosphorylated Rad9 by the apical kinase Ddc2 recruits and activates repair factors such as the signaling kinase Rad53 (human CHK2) to induce a delay of the cell cycle (Schwartz et al., 2002; Sweeney et al., 2005). On the other hand, Rad9 inhibits the DNA end-resection at the DSB sites and the telomere, thereby protecting the broken ends of the DNA and avoiding the occurrence of DNA resection in G1 phase to generate ssDNA (Sweeney et al., 2005; Lazzaro et al., 2008; Clerici et al., 2014; Ferrari et al., 2015).

Chen et al. compared the rate of DNA resection with or without Rad9, showing that the degree of DNA resection was significantly increased in *rad9*Δ cells, with an average rate of 10 kb/h^–1^ compared with 4 kb/h^–1^ in wild-type cells, and moderately increased (6–7 kb/h^–1^) in hta1/2-S129^∗^ or *dot1* mutant cells. It was demonstrated that Rad9 could indeed inhibit DNA end-resection. Conversely, the rate of DNA resection in *Fun30* cells was significantly decreased. Surprisingly, Fun30 was found to be dispensable for resection in the absence of Rad9 (Chen X. et al., 2012). These results suggested that Fun30 is particularly important for remodeling and resection within Rad9-bound chromatin. One possibility is that Fun30 helps to overcome the resection barrier formed by Rad9. Indeed, more Rad9 was accumulated at DSB ends in *fun30*Δ cells than in wild-type ones (Chen X. et al., 2012).

Rad9 is also conserved in various species, and the homolog in humans is 53BP1. 53BP1 is a really important regulator for DSB repair. It was first discovered as a binding partner of the tumor suppressor protein p53 about two decades ago (Iwabuchi et al., 1994). 53BP1 is recruited to DSB sites through recognizing H2AK15Ub and H4K20me2 with its UDR domain and Tudor domain, respectively (Fradet-Turcotte et al., 2013; Wilson et al., 2016). In addition, the recruitment of 53BP1 may be also dependent on the interaction of its BRCT domain at the C-terminus with p53 and EXPAND1 (Huen et al., 2010). Similar to Rad9, 53BP1 has two main functions. First, it acts as a scaffold for DNA damage response factors, promoting the activation of DNA damage checkpoint signals. Second, it inhibits the DNA end-resection and promotes the repair process of non-homologous end joining (Panier and Boulton, 2014).

Densham et al. (2016) found that the double knockout of 53BP1 and SMARCAD1 did make the rates of DNA end-resection similar to WT. This meant that SMARCAD1-direct regulation in processes such as DNA end-resection and Rad51 nuclear foci is dependent on the existence of 53BP1. Moreover, the knockout of SMARCAD1 caused 53BP1 accumulation at the DSB sites in the S phase. These results indicated that SMARCAD1 antagonizes 53BP1 to facilitate DNA end-resection, a regulatory mechanism similar to Fun30 in yeast (Densham et al., 2016).

To address the question of how SMARCAD1^Fun30^ removes 53BP1^Rad9^, Bantele et al. demonstrated the interaction between phosphorylated Fun30 and the scaffold protein Dbp11 through yeast two-hybrid, co-immunoprecipitation and *in vitro* pull-down assay. Mutations of the phosphorylation site affected the recruitment of Fun30, further confirming that Fun30 recruitment to the DSB sites is dependent on scaffold protein Dbp11 and 9-1-1 complex (Bantele et al., 2017). Moreover, they demonstrated that this regulatory process is also conserved in humans. The recruitment of SMARCAD1 to DSB sites is also dependent on CDK-catalyzed phosphorylation on its N-terminus and then binding to TOPBP1 (Dbp11 homolog in humans) (Bantele et al., 2017). Coincidentally, the phosphorylated Fun30 by CDK was recruited through the BRCT1+2 domain of Dbp11, which is also the binding site for Rad9 recruitment. Besides, as a chromatin remodeler, Fun30 has been shown to interact with nucleosomes *in vitro* (Awad et al., 2010). Therefore, Bantele et al. (2017) proposed a regulatory model that Fun30 antagonizes the binding of Rad9 at the DSB sites by occupying the two binding sites of nucleosomes and Dbp11. However, this antagonism is dependent on its chromatin remodeling enzyme activity, because mutations in the active site of ATPase, whether in humans or yeast, caused DNA end-resection defects (Chen X. et al., 2012; Chakraborty et al., 2018).

In summary, the core function of SMARCAD1^Fun30^ in promoting DNA resection is to release the inhibition of 53BP1^Rad9^. SMARCAD1^Fun30^ antagonizes the inhibition effect of 53BP1^Rad9^ by altering the structure or composition of chromatin through its remodeling enzyme activity, thereby promoting the long-range DNA end-resection.

## Molecular Mechanism of SMARCAD1^Fun30^ Promoting the DNA End-Resection on Double-Strand Break Ends

In the past decade, many experimental studies have demonstrated that SMARCAD1^Fun30^ antagonizes the DNA end-resection inhibitor 53BP1^Rad9^ and then promotes the process of homologous recombination. To understand the molecular mechanism of SMARCAD1^Fun30^ promoting the DNA end-resection, mechanistic studies have been carried out on three aspects. First, the recruitment mechanism of SMARCAD1^Fun30^. Second, the factors that regulate the remodeling enzyme activity of SMARCAD1^Fun30^. Finally, the possible remodeling mechanism of SMARCAD1^Fun30^.

### Recruitment Mechanism of SMARCAD1^Fun30^

**FIGURE 3:**
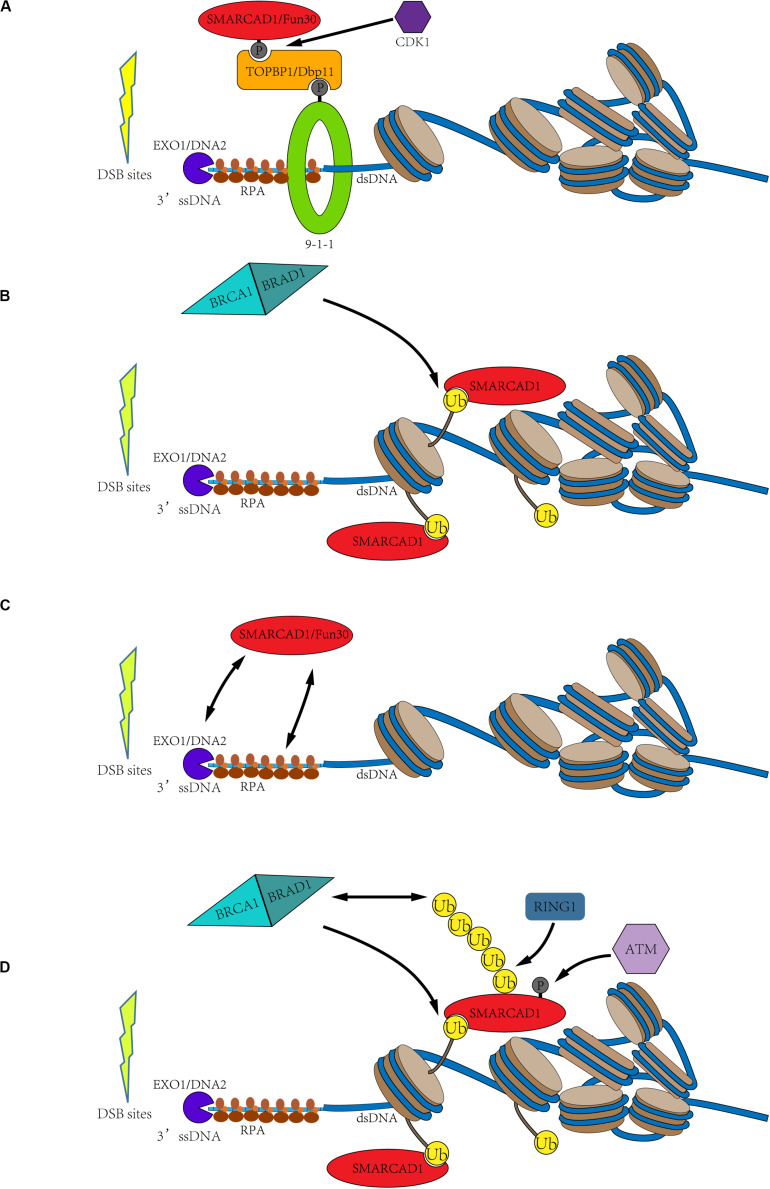
Possible recruitment mechanism of SMARCAD1^Fun30^. **(A)** Cell cycle-dependent SMARCAD1^Fun30^ targeting via the 9-1-1-TOPBP1^Dbp11^-SMARCAD1^Fun30^ complex. The CDK-dependent SMARCAD1^Fun30^ phosphorylation is recognized by the BRCT1+2 domain of TOPBP1^Dbp11^ to form a ternary complex with the 9-1-1 and then recruited to the DSB sites. **(B)** Histone ubiquitination recruits SMARCAD1. SMARCAD1 recognizes BRCA1-BARD1-dependent H2A (K125/127/129) ubiquitination through its CUE domain and then is recruited to DSB sites. **(C)** The recruitment of Fun30 through protein-protein interactions with exonuclease (Exo1), endonuclease Dna2, and single-chain binding protein RPA. It probably constitutes a positive biological feedback loop. **(D)** The C-terminal phosphorylation and ubiquitination of SMARCAD1 facilitate its recruitment. SMARCAD1 is phosphorylated by ATM kinase at T906 residue to promote the RING1-catalyzed K63-polyubiquitination at K905 residue, which recruits BRCA1 to further ubiquitinate histone H2A. Then, the ubiquitinated H2A-containing nucleosomes recruit SMARCAD1. It also constitutes a positive feedback loop.

To elucidate the detailed molecular mechanism by which SMARCAD1^Fun30^ functions at the DNA double-strand break sites, it is necessary to understand how SMARCAD1^Fun30^ is recruited to DSB sites. There are divergent opinions on the recruitment mechanism of SMARCAD1^Fun30^, which are mainly divided into the following four parts (Figure 3).

#### N-Terminus Phosphorylation-Dependent SMARCAD1^Fun30^ Recruitment

Both SMARCAD1 and Fun30 have been identified as substrates of CDK-dependent phosphorylation. Phosphorylation of Fun30 occurs at three residues (S20, S28, and S34), while SMARCAD1 is phosphorylated at T71 residue (Ubersax et al., 2003; Chen X. et al., 2016; Bantele et al., 2017). Phosphorylation has been demonstrated to facilitate the interaction between Fun30 and the scaffold protein Dbp11. This phosphorylation directed interaction might be conserved in species. In humans, the recruitment of SMARCAD1 to DSB sites by binding to TOPBP1 (Dbp11 in yeast) is also dependent on its N-terminal phosphorylation. Then, the interaction between SMARCAD1^Fun30^ and TOPBP1^Dbp11^ results in the formation of a ternary complex with the 9-1-1 complex, thereby targeting SMARCAD1^Fun30^ to the DSB sites. The formation of this ternary complex is essential for the recruitment and function of SMARCAD1^Fun30^ because mutations in the phosphorylation site of SMARCAD1^Fun30^ led to defects in DNA resection (Figure 3A; Bantele et al., 2017).

#### SMARCAD1 Is Recruited to DSB Sites by Binding Ubiquitinated Nucleosome

Another hypothesis is that SMARCAD1^Fun30^ is recruited to the DSB sites by recognizing ubiquitinated nucleosomes with its CUE domain. Fun30 and its orthologs have been bioinformatically predicted to contain at least one CUE domain, especially, SMARCAD1 has two CUE domains (Neves-Costa et al., 2009). The CUE domain has been identified to recognize ubiquitin for many years (Kang et al., 2003; Umebayashi, 2003). Densham et al. confirmed the interaction of SMARCAD1 with H2AUb-containing nucleosomes by *in vitro* pull-down experiments (Densham et al., 2016). Through fluorescence electron microscopy, it was found that mutations of the CUE domain indeed led to a decrease in foci of SMARCAD1 at the DSB sites. Moreover, the transfection of the E3 ligase-inactivating BARD1-R99E mutant into the *BARD1* deletion cells still impaired DNA end resection, and, as mentioned previously, the cells were hypersensitivity to genotoxic agents such as olaparib. However, the expression of a ubiquitin-H2A fusion protein in *BARD1*Δ cells helped to restore DNA end resection and resisted genotoxic agents in a SMARCAD1-dependent manner (Densham et al., 2016). Therefore, SMARCAD1 may recognize BRCA1-BARD1-catalyzed histone H2A (K125/127/129) ubiquitination through its CUE domain to locate at the DSB sites and promote the DNA end-resection (Figure 3B).

However, such a mechanism is unlikely to be an evolutionary conserved (Chen X. et al., 2012; Pfander and Bantele, 2019). First, the ubiquitinated protein specifically recognized by the CUE domain of yeast Fun30 has not been discovered. Second, the mutation of the CUE domain of Fun30 neither affected DNA binding nor DNA end resection (Chen X. et al., 2012). Third, previous studies revealed that SMARCAD1 interacts with the transcriptional repressor KAP1 through its CUE domain to target heterochromatin regions (Rowbotham et al., 2011). Ding et al. (2018) discovered that KAP1 interacts with the CUE1 domain through its RBCC domain, promoting the recruitment of SMARCAD1 to the characteristic genes. The molecular details of the interaction between the RBCC domain and CUE1 domain were recently elucidated by Svejstrup and co-workers (Lim et al., 2019). SMARCAD1 interacts with the coiled-coil surface of the RBCC domain through its CUE1 domain. Although the structure of the RBCC domain and the ubiquitin are completely different, the affinity of the CUE1 domain of SMARCAD1 for the KAP1 RBCC domain (Kd≈158 nM) is over 1,000-fold greater than that for mono-Ub (Kd≈952 μM). These above results suggested that the CUE domain of Fun30 and the CUE1 domain of SMARCAD1 may not contribute to its recruitment to the DSB sites.

53BP1 is recruited to the DSB sites through bivalent recognition of ubiquitinated/methylated nucleosomes, which is completely different from the recognition of phosphorylated/methylated nucleosomes by Rad9 in yeast (Fradet-Turcotte et al., 2013; Wilson et al., 2016). Therefore, the recruitment mechanism that recognizes ubiquitinated nucleosomes through the second CUE domain may be the result of species evolution. Although there is already evidence *in vivo* that the CUE domain is essential for the recruitment of SMARCAD1 and DNA end-resection, the *in vitro* reconstruction experiments followed by the structural determination of the related complex may still be required to support this theory. Nonetheless, several post-translational modifications, such as acetylation and methylation, have been demonstrated to regulate the recruitment and function of chromatin remodelers (Javaheri et al., 2006; Wysocka et al., 2006; VanDemark et al., 2007), but SMARCAD1 related process is the first case in linking ubiquitination to the regulation of chromatin remodeler.

#### Fun30 Is Recruited to DSB Sites by Interacting With Exo1, Dna2, and RPA

In addition to the two possible recruitment mechanisms mentioned above, Fun30 may also be recruited to DSB sites by nucleases and ssDNA binding protein RPA. Biochemical experiments demonstrated that Fun30 co-immunoprecipitated with some DNA resection factors, such as exonuclease Exo1, endonuclease Dna2, and single-chain binding protein RPA. Moreover, further evidence showed that the resection enzymes were recruited next to DSB ends but failed to spread efficiently farther from the break site in *fun30*Δ cells (Chen X. et al., 2012). Therefore, these may constitute a positive feedback loop, in which Fun30 promotes the DNA end-resection, and then DNA resection factors promote the recruitment of Fun30, further promoting the DNA-end resection (Figure 3C).

#### The C-Terminal Phosphorylation and Ubiquitination of SMARCAD1 Facilitate Its Recruitment

As mentioned above, SMARCAD1 is a potential substrate of ATR and ATM (Matsuoka et al., 2007). Later, Chakraborty et al. (2018) confirmed that SMARCAD1 is phosphorylated at T906 residue by ATM and this modification is essential for DNA end-resection. T906A mutation was demonstrated to seriously affect the process of DNA end-resection, but phosphorylation mimic mutation (T906E) had little effect. This study also revealed that T906 phosphorylation promotes RING1-dependent K905 ubiquitination, mainly forming the K63 ubiquitin chain. Both T906A and K905R mutations affected the accumulation of SMARCAD1 at the DSB sites. Surprisingly, these mutations also impaired the accumulation of BRCA1 at the DSB sites. Therefore, Chakraborty et al. speculated that T906 phosphorylation and the K63-polyubiquitination at K905 residue facilitate the recruitment of BRCA1, thereby blocking 53BP1 recruitment to DSB sites (Chakraborty et al., 2018). These also constitute a positive feedback loop. SMARCAD1 is phosphorylated by ATM to promote the RING1-dependent K63-polyubiquitination at K905 residue, which recruits BRCA1 to DSB sites. Subsequently, BRCA1, as an E3 ligase, could ubiquitinate the C-terminal of histone H2A, thereby further promoting the recruitment of SMARCAD1 (Figure 3D).

Based on the reported studies, the most likely recruitment mechanism is that SMARCAD1^Fun30^ is phosphorylated by CDK and then recruited to the DSB sites through the 9-1-1 complex and a scaffold protein TOPBP1^Dbp11^. And this recruitment mechanism is cell cycle-dependent and highly conserved among species. However, it is confusing that the mutation of the N-terminal phosphorylation site of SMARCAD1^Fun30^ did not affect its recruitment near the break site (approximately 1 kb), but significantly impaired the recruitment at the far end (Chakraborty et al., 2018). This phenomenon may indicate that SMARCAD1^Fun30^ has other initial recruitment mechanisms at the near end of the DSB. In general, the recruitment of SMARCAD1^Fun30^ may be the result of a combination of factors, which need to be further studied.

### Activity Regulation

To function properly, the enzyme activity of chromatin remodelers is always regulated by their domains or other interacting proteins. Chromatin remodelers generally have five characteristics, including (Clapier et al., 2017)

(a) the ability to bind nucleosomes;
(b) a single catalytic subunit that contains an ATPase domain;
(c) domains and/or subunits that interact with chaperones, site-specific transcription factors or other chromatin proteins;
(d) domains and/or subunits that recognize histone post-translational modifications;
(e) domains and/or subunits that regulate the ATPase domain.

The first two properties listed above enable chromatin remodelers to act on nucleosomes, whereas the other three attributes allow them to selectively act on the nucleosomes at specific locations (Clapier et al., 2017). Among them, histone modifications act as an important epigenetic regulator to be highly associated with chromatin remodelers. Typically, histone modifications will affect the targeting of chromatin remodelers to specific nucleosomes. Almost all remodelers have domains or subunits that recognize specific histone post-translational modifications. For example, the *Drosophila melanogaster* nucleosome remodeling factor (NURF) remodeling complex binds to Lys4 trimethylated histone H3 (H3K4me3) (Javaheri et al., 2006; Wysocka et al., 2006). Similarly, the tandem bromodomain of the subunit Rsc4 in the RSC complex specifically recognizes Lys14 acetylated histone H3 (H3K14ac), and this single acetylated residue is sufficient to enhance RSC binding to nucleosomes *in vitro* (VanDemark et al., 2007).

More importantly, histone modifications can directly regulate the ATPase activity of chromatin remodelers through an allosteric activation mechanism. For the SWI/SNF subfamily members, site-specific H3 acetylation was shown to enhance the remodeling activities of yeast RSC and SWI/SNF, without increasing their nucleosome-binding affinities (Ferreira et al., 2007; Chatterjee et al., 2011). And H3K64ac can increase the sliding activity of Chd1 (Di Cerbo et al., 2014).

This poses an interesting question as to whether the activity of SMARCAD1 is also regulated by histone modifications or specific chromatin structures at the DSB sites.

#### H2A C-Terminal Ubiquitination Regulates SMARCAD1 Activity

It was mentioned above that SMARCAD1 can recognize the ubiquitinated nucleosome and then be recruited to the DSB sites (Densham et al., 2016; Uckelmann et al., 2018). Mutations of the CUE domain will affect the function of SMARCAD1 in promoting the DNA end-resection. However, the mutation of the CUE domain did not completely reduce the foci of SMARCAD1 at DSB sites. These results implied that the function of SMARCAD1 regulated by ubiquitination might be not limited to the recruitment mechanism. H2A C-terminal ubiquitination (K125/127/129) may participate in the regulation of the enzyme activity of SMARCAD1. Certainly, further evidence of H2A ubiquitination-dependent SMARCAD1 activation should be provided, especially the precise *in vitro* reconstitution experiments may give intuitive results. Moreover, the structural basis of H2A ubiquitination-activated remodeling may also be an interesting question.

#### ssDNA Regulates SMARCAD1^Fun30^ Activity

*In vitro*, Fun30 was demonstrated to directly bind naked DNA, nucleosome, and chromatin (Awad et al., 2010; Adkins et al., 2013). Furthermore, the gel-shift experiment showed that Fun30 has a stronger bind ability to 0W47 NCP than 0W0 NCP (“W” corresponds to Widom 601 nucleosome-positioning sequence and the numbers indicate the length of linker DNA on both sides of the 601 DNA). Therefore, Fun30 may have a linker DNA binding pattern similar to SWR1C. Surprisingly, the ATPase activity of Fun30 stimulated by single-stranded DNA is 25 times more than that stimulated by double-stranded one (Adkins et al., 2017).

Based on these results, we hypothesize that ssDNA may regulate the activity of SMARCAD1. At the beginning of the DNA end-resection, DSB ends are nucleolytically digested from the DSB sites to generate 3′ ssDNA. In the unresected place, the DNA is wrapped around the octamer, which will form the dsDNA-NCP-ssDNA hybrid. In this hybrid, ssDNA flanking the nucleosome may also promote the binding and activity of Fun30, which helps SMARCAD1^Fun30^ to disrupt the nucleosome organization around DSB and facilitate the role of DNA resection factors.

#### C-Terminal Phosphorylation Regulates SMARCAD1 Activity

It was demonstrated that phosphorylation of SMARCAD1 at T906 residue is necessary for promoting DNA end-resection. T906A mutation significantly affected the process of DNA end-resection, but phosphorylation mimic mutations (T906E) had little effect (Chakraborty et al., 2018). Coincidentally, the T906 residue locates on the helicase domain of SMARCAD1 (aa 858-1010), which is the key position of SMARCAD1 for interacting with nucleosomes to disrupt DNA-histone interactions. Therefore, it is reasonable to speculate that the phosphorylation of T906 at the C-terminus of SMARCAD1 may participate in the SMARCAD1-directed nucleosome remodeling.

### Remodeling Mechanism

It can be concluded from the previous studies that SMARCAD1^Fun30^ removes 53BP1^Rad9^ by competing with the complex formation between nucleosome and TOPBP1^Dbp11^, thereby promoting the DNA end-resection. This does not seem to require the chromatin remodeling activity of SMARCAD1^Fun30^. However, the ATPase activity of the remodeling enzyme is also necessary to promote the DNA end resection, indicating that the competition between SMARCAD1^Fun30^ and 53BP1^Rad9^ is not the only source of antagonism. Therefore, SMARCAD1^Fun30^ should alter the structure of chromatin or change the composition of chromatin through its remodeling enzyme activity to antagonize the inhibition effect of 53BP1^Rad9^. For example, some studies have shown that the removal of the phosphorylation mark of γH2A would lead to a defect in the Rad9 chromatin association (Javaheri et al., 2006; Hammet et al., 2007; Eapen et al., 2012; Clerici et al., 2014), which is consistent with the H2A-H2B dimer exchange activity of Fun30 (Awad et al., 2010). It poses an interesting question of how SMARCAD1^Fun30^ utilizes its chromatin remodeling activity to release the occupation of 53BP1^Rad9^ and promote the DNA end-resection.

However, there is no clear conclusion about the remodeling mechanism of SMARCAD1^Fun30^ in the process of DNA damage repair, but several studies have been conducted to address this question. First, as mentioned above, Fun30 was found to share a high degree of homology with Swr1 and Ino80 (Flaus et al., 2006). Swr1 is the core catalytic subunit of the SWR1 complex. SWR1 complex of the INO80 subfamily cannot slide nucleosomes but has the activity for histone dimer exchange. The high-resolution structure of the SWR1 complex bound to a nucleosome revealed that Swr1 binding caused the DNA to produce a 1 bp bugle at SHL2. Due to the single-base pair DNA translocation, the interaction between the N-terminal domain of SWR1 and another circle of DNA wrapping around the nucleosome is strengthened, which limits the further sliding of the nucleosome (Willhoft et al., 2018). This may be the reason why SWR1 is unable to slide nucleosomes. Unlike the SWR1 complex, the INO80 complex can not only slide but also edit a nucleosome (Brahma et al., 2017). Nevertheless, both INO80 and SWR1 can cause DNA unwrapping and then disrupt the interaction between the DNA and H2A-H2B dimers (Ayala et al., 2018; Willhoft et al., 2018). Therefore, Fun30 may have a similar remodeling mechanism of histone dimer exchange. Second, *in vitro* experiments indicated that the activity of Fun30 for H2A-H2B dimer exchange is far stronger than that for sliding nucleosomes (Awad et al., 2010). Third, knockout of Fun30 was unable to affect the positioning of nucleosomes near the DSB sites *in vivo*. Of course, this phenomenon may also be caused by the inability of current technology to dynamically observe changes in the position of nucleosomes in DNA damage repair sites (Costelloe et al., 2012). Fourth, Fun30 has a particular binding preference, it is more inclined to bind nucleosomes without phosphorylation rather than the H2AS129 phosphorylated nucleosomes (Eapen et al., 2012). This phenomenon may imply that Fun30 removes the γH2A-H2B marker through the activity of the H2A/H2B dimer exchange, thereby removing the occupation of Rad9 at DSB sites.

Based on these results, we propose a possible remodeling mechanism of Fun30. The model is that Fun30 removes γH2AX-H2B dimer and then replaces it with other histone dimers (such as H2A.Z-H2B) through its dimer exchange activity, thereby releasing the occupation of Rad9 at DSB sites. Some experimental results showed that the distribution of H2A.Z is indeed affected by Fun30 and Fft3, whether in the whole genome or centromeres, proximal enantiomers, and subtelomere (Stralfors et al., 2011; Durand-Dubief et al., 2012). Moreover, many studies have demonstrated that H2A.Z-H2B plays an important role in DNA damage repair (van Attikum et al., 2007; Kalocsay et al., 2009; Xu et al., 2012; Adkins et al., 2013; Lademann et al., 2017). However, it is unclear whether the change of the H2A.Z distribution caused by Fun30 will occur at the DSB sites. It is also unknown whether the introduction of H2A.Z-H2B contributes to the role of DNA resection factors. Moreover, other experimental data has shown that the occupancy of nucleosomes with H2AS129 phosphorylation at the DNA damage site does not change significantly in *fun30*Δ cells (Eapen et al., 2012). Bantele (2018) suggested that they observed an increase of the γH2A chip enrichment in *fun30*Δ cells over the measured γH2A regions, instead of that in DNA break-proximal regions. Therefore, it remains to be studied whether Fun30 exchanges rH2A-H2B dimer *in vivo*.

**FIGURE 4:**
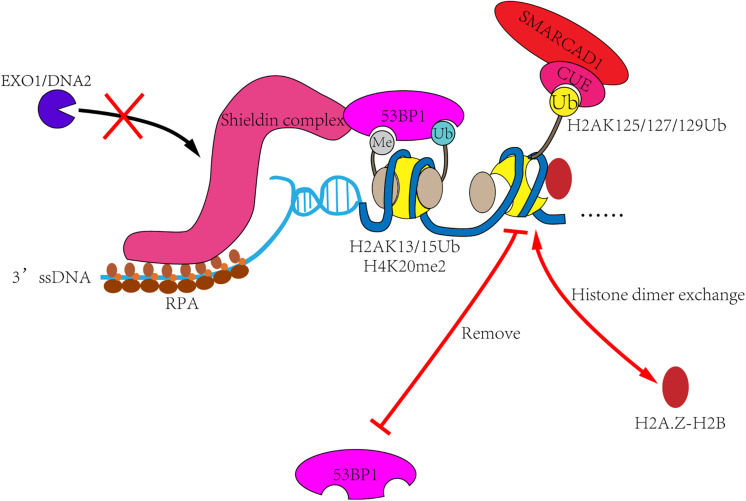
The remodeling mechanism of SMARCAD1^Fun30^. In the G1 phase, 53BP1 and its effector proteins such as Shieldin complex are recruited to DSB sites, then inhibit the DNA end-resection and promote the repair process of non-homologous end-joining. In S and G2 phases, SMARCAD1 is recruited to DSB sites by interacting with TOPBP1 and ubiquitinated nucleosomes. Then SMARCAD1^Fun30^ removes γH2AX-H2B dimer and replace it with other histone dimers, such as H2A.Z-H2B. It will also remove 53BP1 recognized histone markers (such as H2AK13/15Ub), resulting in 53BP1 reposition and allowing the completion of resection.

In humans, the regulatory mechanism is more complex. First, 53BP1 is recruited to the DSB sites through bivalent recognition of H2A15Ub/H4K20me2 (Fradet-Turcotte et al., 2013; Wilson et al., 2016), which is completely different from the recognition of H2AS129ph/H3K79me2 by yeast Rad9 (Wysocki et al., 2005; Toh et al., 2006; Grenon et al., 2007; Hammet et al., 2007). Second, BRCA1 is the second 53BP1 antagonist present in humans. BRCA1 is recruited to the DSB sites in S and G2 phases, lifting the occupation of 53BP1 and starting the process of homologous recombination. BRCA1 primarily functions as an E3-ligase to form histone H2A ubiquitination. As a downstream factor of BRCA1, SMARCAD1 may have evolved to recognizes ubiquitination through the CUE domain, making the regulation more accurate. Finally, 53BP1 can recruit more effector proteins to form complexes, such as RIF3, Shieldin, PTIP, etc. Similar mechanisms have not been described in yeast (Xu et al., 2015; Dev et al., 2018; Findlay et al., 2018; Gupta et al., 2018; Mirman et al., 2018; Noordermeer et al., 2018; Setiaputra and Durocher, 2019). This may also be the reason why more complex ubiquitin-dependent regulation is needed in humans (Figure 4).

## Summary and Outlook

SMARCAD1 and its orthologs, the newly identified chromatin remodelers are highly associated with a variety of cellular processes. For example, SMARCAD1 helps to establish heterochromatin *de novo*, Fft3 stabilizes nucleosomes during replication to maintain the heterochromatin, and SMARCAD1 also promotes the long-range resection by removing the DNA resection inhibitors. Although the molecular mechanisms for the diverse functions of SMARCAD1^Fun30^ need to be further explored, the rising knowledge is deepening our understanding of SMARCAD1^Fun30^-related regulation.

First, the ssDNA is produced during both homologous recombination and DNA replication. Therefore, it is possible that ssDNA can activate the enzyme activity of SMARCAD1^Fun30^. Second, SMARCAD1 binds to PCNA during DNA replication, while it also interacts with the Rad9 subunit of 9-1-1 complex in response to DNA damage repair. Both PCNA and the 9-1-1 complex are composed of three proteins to form a similar circular structure, loading on the ds-ssDNA junction. This phenomenon suggested that the DNA clamp should play an important role in regulating the activity of SMARCAD1^Fun30^ (Pfander and Bantele, 2019).

**FIGURE 5:**
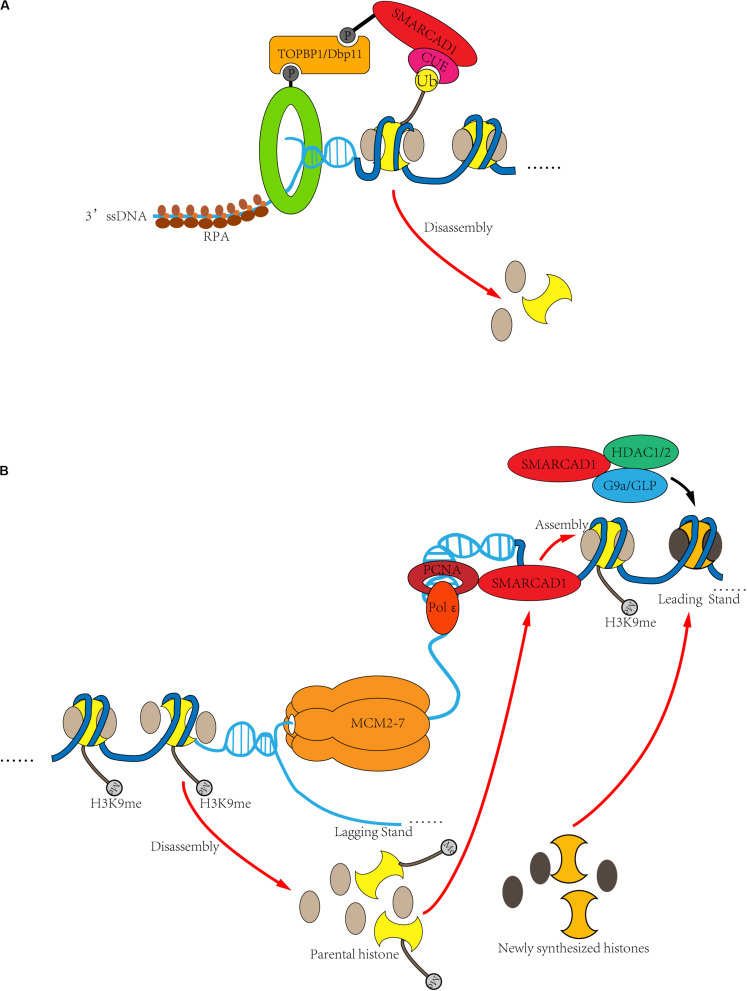
DNA clamp regulates the role of SMARCAD1 in DNA replication and DNA damage repair. **(A)** In response to DSBs, SMARCAD1 promotes long-range DNA end-resection though mobilization and eviction of nucleosomes. When double-strand breaks occur in DNA, the 9-1-1 complex is loaded at the ss-dsDNA junction, and the dsDNA is located upstream of the ss-dsDNA junction. By interacting with the TOPBP1^Dbp11^ and 9-1-1 to form a ternary complex, SMARCAD1 is ideally positioned to remove nucleosomes ahead of the resecting nucleases through its chromatin remodeler activity. Moreover, ubiquitinated H2A appears to stabilize SMARCAD1 at the DSB sites, likely via CUE domain-dependent binding of SMARCAD1 to ubiquitinated H2A. **(B)** During DNA replication, SMARCAD1 contributes to the establishment and maintenance of heterochromatin. SMARCAD1 interacts with PCNA to form a key platform for protein recruitment at the replication fork. PCNA is loaded at the primer-template junction and SMARCAD is located after replisome. In this way, the dsDNA is located downstream of the ss-dsDNA junction. SMARCAD1 can prevent loss of parental histone markers to ensure the inheritance of heterochromatin markers of newly replicated DNA. On the other hand, SMARCAD1 facilitates histone deacetylase HDAC1/2 to deacetylate histone H3/H4 and then histone methylase G9a/GLP to methylate histone H3K9, thus *de novo* establishes the heterochromatin.

The above observations pose an interesting question as to why SMARCAD1^Fun30^ functions to evict nucleosomes during DNA end-resection but stabilizes nucleosomes during DNA replication. Although both DNA replication and DNA damage repair require SMARCAD1 binding with the DNA clamp, the location of SMARCAD1 is completely different in the two processes. During DNA end-resection, the dsDNA is located upstream of the ss-dsDNA junction. Therefore, the 9-1-1 complex is loaded in front of the resection nucleases. By interacting with the 9-1-1 complex, SMARCAD1^Fun30^ is positioned to remove potential obstacles ahead of the resecting nucleases (Figure 5A). PCNA is loaded at the primer-template junction and it moves behind DNA polymerase and helicase. Therefore, the newly synthesized dsDNA is downstream of the ss-dsDNA junction. The interaction between SMARCAD1 and PCNA makes it work after replisome. In this position, SMARCAD1 can stabilize nucleosomes to ensure the inheritance of heterochromatin markers of newly replicated chromatin (Figure 5B). In conclusion, despite the surprising similarities in the function of SMARCAD1 in different cellular processes, the different locations of SMARCAD1 lead to different roles, such as evicting nucleosomes during resection but stabilizing nucleosomes during DNA replication.

Despite these advances in recent years, some questions still need to be further answered, for example, how the remodeling activity of SMARCAD1 is activated by ubiquitinated nucleosomes or ssDNA and how SMARCAD1 removes the obstacles (such as 53BP1 and nucleosomes) through its histone dimer exchange activity. Take advantage of the recent advances in chemical protein synthesis (McGinty et al., 2008; Fang et al., 2011, 2012; Fierz et al., 2011; Siman et al., 2013; Li et al., 2014, 2018; Morgan et al., 2016; Ai et al., 2019; Chu et al., 2019; Pan et al., 2019) and cryo-electron microscopy techniques, the biochemical and structural studies of ubiquitinated nucleosomes have recently received increasing attention (Zhou L. et al., 2016; Anderson et al., 2019; Hsu et al., 2019; Jang et al., 2019; Park et al., 2019; Valencia-Sanchez et al., 2019; Worden and Wolberger, 2019; Worden et al., 2019; Xue et al., 2019; Yao et al., 2019; Dao et al., 2020). Both the *in vitro* reconstruction experiments and the structural analysis of the SMARCAD1-nucleosome complex may provide new insights into the remodeling mechanism of SMARCAD1. Elucidating the molecular mechanisms for functions of SMARCAD1 may be a valuable topic in the following years. Histone ubiquitination-dependent SMARCAD1 recruitment and activation may even lead to comprehension of a new mechanistic model of chromatin remodeling that is regulated by post-translational modifications.

## Author Contributions

Z-BT wrote the manuscript and designed the figures. H-SA and J-BL revised the manuscript. All authors approved the article for publication.

## Conflict of Interest

The authors declare that the research was conducted in the absence of any commercial or financial relationships that could be construed as a potential conflict of interest.

## References

[B1] Adkins N. L. Niu H. Sung P. Peterson C. L. (2013). Nucleosome dynamics regulates DNA processing. *Nat. Struct. Mol. Biol.* 20 836–842. 10.1038/nsmb.2585 23728291 PMC3711194

[B2] Adkins N. L. Swygert S. G. Kaur P. Niu H. Grigoryev S. A. Sung P. (2017). Nucleosome-like, single-stranded DNA (ssDNA)-histone octamer complexes and the implication for DNA double strand break repair. *J. Biol. Chem.* 292 5271–5281. 10.1074/jbc.M117.776369 28202543 PMC5392674

[B3] Adra C. N. Donato J. L. Badovinac R. Syed F. Kheraj R. Cai H. (2000). SMARCAD1, a novel human helicase family-defining member associated with genetic instability: cloning, expression, and mapping to 4q22-q23, a band rich in breakpoints and deletion mutants involved in several human diseases. *Genomics* 69 162–173. 10.1006/geno.2000.6281 11031099

[B4] Ai H. Guo Y. Sun D. Liu S. Qi Y. Guo J. (2019). Examination of the deubiquitylation site selectivity of USP51 by using chemically synthesized ubiquitylated histones. *ChemBioChem* 20 221–229. 10.1002/cbic.201800432 30192049

[B5] Al Kubaisy E. Arafat K. De Wever O. Hassan A. H. Attoub S. (2016). SMARCAD1 knockdown uncovers its role in breast cancer cell migration, invasion, and metastasis. *Exp. Opin. Ther. Targets* 20 1035–1043. 10.1080/14728222.2016.1195059 27232533

[B6] Anderson C. J. Baird M. R. Hsu A. Barbour E. H. Koyama Y. Borgnia M. J. (2019). Structural basis for recognition of ubiquitylated nucleosome by Dot1L methyltransferase. *Cell Rep.* 26 1681–1690.e5. 10.1016/j.celrep.2019.01.058 30759380 PMC6392056

[B7] Arafat K. Al Kubaisy E. Sulaiman S. Karam S. M. Al Natour Z. Hassan A. H. (2018). SMARCAD1 in breast cancer progression. *Cell Physiol. Biochem.* 50 489–500. 10.1159/000494163 30308496

[B8] Awad S. Ryan D. Prochasson P. Owen-Hughes T. Hassan A. H. (2010). The Snf2 homolog Fun30 acts as a homodimeric ATP-dependent chromatin-remodeling enzyme. *J. Biol. Chem.* 285 9477–9484. 10.1074/jbc.M109.082149 20075079 PMC2843198

[B9] Ayala R. Willhoft O. Aramayo R. J. Wilkinson M. McCormack E. A. Ocloo L. (2018). Structure and regulation of the human INO80-nucleosome complex. *Nature* 556 391–395. 10.1038/s41586-018-0021-6 29643506 PMC5937682

[B10] Bantele S. C. Ferreira P. Gritenaite D. Boos D. Pfander B. (2017). Targeting of the Fun30 nucleosome remodeller by the Dpb11 scaffold facilitates cell cycle-regulated DNA end resection. *Elife* 6:e21687. 10.7554/eLife.21687 28063255 PMC5300703

[B11] Bantele S. C. S. (2018). *Cell Cycle-Regulated Signaling and Remodeling at DNA Double-Strand Breaks.* Munich: LMU.

[B12] Barbaric S. Luckenbach T. Schmid A. Blaschke D. Horz W. Korber P. (2007). Redundancy of chromatin remodeling pathways for the induction of the yeast PHO5 promoter *in vivo*. *J. Biol. Chem.* 282 27610–27621. 10.1074/jbc.M700623200 17631505

[B13] Bartholomew B. (2014). Regulating the chromatin landscape: structural and mechanistic perspectives. *Annu. Rev. Biochem.* 83 671–696. 10.1146/annurev-biochem-051810-093157 24606138 PMC4332854

[B14] Brahma S. Udugama M. I. Kim J. Hada A. Bhardwaj S. K. Hailu S. G. (2017). INO80 exchanges H2A.Z for H2A by translocating on DNA proximal to histone dimers. *Nat. Commun*. 8:15616. 10.1038/ncomms15616 28604691 PMC5472786

[B15] Byeon B. Wang W. Barski A. Ranallo R. T. Bao K. Schones D. E. (2013). The ATP-dependent chromatin remodeling enzyme Fun30 represses transcription by sliding promoter-proximal nucleosomes. *J. Biol. Chem.* 288 23182–23193. 10.1074/jbc.M113.471979 23779104 PMC3743490

[B16] Chakraborty S. Pandita R. K. Hambarde S. Mattoo A. R. Charaka V. Ahmed K. M. (2018). SMARCAD1 phosphorylation and ubiquitination are required for resection during DNA double-strand break repair. *iScience* 2 123–135. 10.1016/j.isci.2018.03.016 29888761 PMC5993204

[B17] Chatterjee N. Sinha D. Lemma-Dechassa M. Tan S. Shogren-Knaak M. A. Bartholomew B. (2011). Histone H3 tail acetylation modulates ATP-dependent remodeling through multiple mechanisms. *Nucleic Acids Res.* 39 8378–8391. 10.1093/nar/gkr535 21749977 PMC3201869

[B18] Chen X. Cui D. Papusha A. Zhang X. Chu C. D. Tang J. (2012). The Fun30 nucleosome remodeller promotes resection of DNA double-strand break ends. *Nature* 489 576–580. 10.1038/nature11355 22960743 PMC3640768

[B19] Chen X. Niu H. Yu Y. Wang J. Zhu S. Zhou J. (2016). Enrichment of Cdk1-cyclins at DNA double-strand breaks stimulates Fun30 phosphorylation and DNA end resection. *Nucleic Acids Res.* 44 2742–2753. 10.1093/nar/gkv1544 26801641 PMC4824098

[B20] Chen Z. Tran M. Tang M. Wang W. Gong Z. Chen J. (2016). Proteomic analysis reveals a novel mutator S (MutS) partner involved in mismatch repair pathway. *Mol. Cell Proteomics* 15 1299–1308. 10.1074/mcp.M115.056093 27037360 PMC4824856

[B21] Chu G.-C. Pan M. Li J. Liu S. Zuo C. Tong Z.-B. (2019). Cysteine-aminoethylation-assisted chemical ubiquitination of recombinant histones. *J. Am. Chem. Soc.* 141 3654–3663. 10.1021/jacs.8b13213Clapier 30758956

[B22] Clapier C. R. Cairns B. R. (2009). The biology of chromatin remodeling complexes. *Annu. Rev. Biochem.* 78 273–304. 10.1146/annurev.biochem.77.062706.153223 19355820

[B23] Clapier C. R. Iwasa J. Cairns B. R. Peterson C. L. (2017). Mechanisms of action and regulation of ATP-dependent chromatin-remodelling complexes. *Nat. Rev. Mol. Cell Biol.* 18 407–422. 10.1038/nrm.2017.26 28512350 PMC8127953

[B24] Clerici M. Trovesi C. Galbiati A. Lucchini G. Longhese M. P. (2014). Mec1/ATR regulates the generation of single-stranded DNA that attenuates Tel1/ATM signaling at DNA ends. *EMBO J.* 33 198–216. 10.1002/embj.201386041 24357557 PMC3989615

[B25] Costelloe T. Louge R. Tomimatsu N. Mukherjee B. Martini E. Khadaroo B. (2012). The yeast Fun30 and human SMARCAD1 chromatin remodellers promote DNA end resection. *Nature* 489 581–584. 10.1038/nature11353 22960744 PMC3493121

[B26] Dao H. T. Dul B. E. Dann G. P. Liszczak G. P. Muir T. W. (2020). A basic motif anchoring ISWI to nucleosome acidic patch regulates nucleosome spacing. *Nat. Chem. Biol.* 16 134–142. 10.1038/s41589-019-0413-4 31819269 PMC6982587

[B27] Densham R. M. Garvin A. J. Stone H. R. Strachan J. Baldock R. A. Daza-Martin M. (2016). Human BRCA1–BARD1 ubiquitin ligase activity counteracts chromatin barriers to DNA resection. *Nat. Struct. Mol. Biol.* 23 647–655. 10.1038/nsmb.3236 27239795 PMC6522385

[B28] Dev H. Chiang T.-W. W. Lescale C. de Krijger I. Martin A. G. Pilger D. (2018). Shieldin complex promotes DNA end-joining and counters homologous recombination in BRCA1-null cells. *Nat. Cell Biol.* 20 954–965. 10.1038/s41556-018-0140-1 30022119 PMC6145444

[B29] Di Cerbo V. Mohn F. Ryan D. P. Montellier E. Kacem S. Tropberger P. (2014). Acetylation of histone H3 at lysine 64 regulates nucleosome dynamics and facilitates transcription. *Elife* 3:e01632. 10.7554/eLife.01632.001 24668167 PMC3965291

[B30] Ding D. Bergmaier P. Sachs P. Klangwart M. Ruckert T. Bartels N. (2018). The CUE1 domain of the SNF2-like chromatin remodeler SMARCAD1 mediates its association with KRAB-associated protein 1 (KAP1) and KAP1 target genes. *J. Biol. Chem.* 293 2711–2724. 10.1074/jbc.RA117.000959 29284678 PMC5827453

[B31] Doiguchi M. Nakagawa T. Imamura Y. Yoneda M. Higashi M. Kubota K. (2016). SMARCAD1 is an ATP-dependent stimulator of nucleosomal H2A acetylation via CBP, resulting in transcriptional regulation. *Sci. Rep.* 6:20179. 10.1038/srep20179 26888216 PMC4757861

[B32] Durand-Dubief M. Will W. R. Petrini E. Theodorou D. Harris R. R. Crawford M. R. (2012). SWI/SNF-like chromatin remodeling factor Fun30 supports point centromere function in *S. cerevisiae*. *PLoS Genet.* 8:e1002974. 10.1371/journal.pgen.1002974 23028372 PMC3459985

[B33] Eapen V. V. Sugawara N. Tsabar M. Wu W. H. Haber J. E. (2012). The *Saccharomyces cerevisiae* chromatin remodeler Fun30 regulates DNA end resection and checkpoint deactivation. *Mol. Cell. Biol.* 32 4727–4740. 10.1128/mcb.00566-12 23007155 PMC3486187

[B34] Eustermann S. Schall K. Kostrewa D. Lakomek K. Strauss M. Moldt M. (2018). Structural basis for ATP-dependent chromatin remodelling by the INO80 complex. *Nature* 556 386–390. 10.1038/s41586-018-0029-y 29643509 PMC6071913

[B35] Fang G. M. Li Y. M. Shen F. Huang Y. C. Li J. B. Lin Y. (2011). Protein chemical synthesis by ligation of peptide hydrazides. *Angew. Chem. Int. Ed.* 50 7645–7649. 10.1002/anie.201100996 21648030

[B36] Fang G. M. Wang J. X. Liu L. (2012). Convergent chemical synthesis of proteins by ligation of peptide hydrazides. *Angew. Chem. Int. Ed.* 51 10347–10350. 10.1002/anie.201203843 22968928

[B37] Farnung L. Vos S. M. Wigge C. Cramer P. (2017). Nucleosome-Chd1 structure and implications for chromatin remodelling. *Nature* 550 539–542. 10.1038/nature24046 29019976 PMC5697743

[B38] Feng Y. Tian Y. Wu Z. Xu Y. (2018). Cryo-EM structure of human SRCAP complex. *Cell Res.* 28 1121–1123. 10.1038/s41422-018-0102-y 30337683 PMC6218446

[B39] Ferrari M. Dibitetto D. De Gregorio G. Eapen V. V. Rawal C. C. Lazzaro F. (2015). Functional interplay between the 53BP1-ortholog Rad9 and the Mre11 complex regulates resection, end-tethering and repair of a double-strand break. *PLoS Genet.* 11:e1004928. 10.1371/journal.pgen.1004928 25569305 PMC4287487

[B40] Ferreira H. Flaus A. Owen-Hughes T. (2007). Histone modifications influence the action of Snf2 family remodelling enzymes by different mechanisms. *J. Mol. Biol.* 374 563–579. 10.1016/j.jmb.2007.09.059 17949749 PMC2279226

[B41] Fierz B. (2014). Synthetic chromatin approaches to probe the writing and erasing of histone modifications. *ChemMedChem* 9 495–504. 10.1002/cmdc.201300487 24497444

[B42] Fierz B. Chatterjee C. McGinty R. K. Bar-Dagan M. Raleigh D. P. Muir T. W. (2011). Histone H2B ubiquitylation disrupts local and higher-order chromatin compaction. *Nat. Chem. Biol.* 7 113–119. 10.1038/nchembio.501 21196936 PMC3078768

[B43] Filion G. J. van Bemmel J. G. Braunschweig U. Talhout W. Kind J. Ward L. D. (2010). Systematic protein location mapping reveals five principal chromatin types in *Drosophila* cells. *Cells* 143 212–224. 10.1016/j.cell.2010.09.009 20888037 PMC3119929

[B44] Findlay S. Heath J. Luo V. M. Malina A. Morin T. Coulombe Y. (2018). SHLD2/FAM35A co-operates with REV7 to coordinate DNA double-strand break repair pathway choice. *EMBO J.* 37:e100158. 10.15252/embj.2018100158 30154076 PMC6138439

[B45] Flaus A. Martin D. M. Barton G. J. Owen-Hughes T. (2006). Identification of multiple distinct Snf2 subfamilies with conserved structural motifs. *Nucleic Acids Res.* 34 2887–2905. 10.1093/nar/gkl295 16738128 PMC1474054

[B46] Fradet-Turcotte A. Canny M. D. Escribano-Díaz C. Orthwein A. Leung C. C. Huang H. (2013). 53BP1 is a reader of the DNA-damage-induced H2A Lys 15 ubiquitin mark. *Nature* 499 50–54. 10.1038/nature12318 23760478 PMC3955401

[B47] Granata M. Lazzaro F. Novarina D. Panigada D. Puddu F. Abreu C. M. (2010). Dynamics of Rad9 chromatin binding and checkpoint function are mediated by its dimerization and are cell cycle–regulated by CDK1 activity. *PLoS Genet.* 6:e1001047. 10.1371/journal.pgen.1001047 20700441 PMC2916856

[B48] Grenon M. Costelloe T. Jimeno S. O’Shaughnessy A. FitzGerald J. Zgheib O. (2007). Docking onto chromatin via the *Saccharomyces cerevisiae* Rad9 Tudor domain. *Yeast* 24 105–119. 10.1002/yea.1441 17243194

[B49] Gupta R. Somyajit K. Narita T. Maskey E. Stanlie A. Kremer M. (2018). DNA repair network analysis reveals shieldin as a key regulator of NHEJ and PARP inhibitor sensitivity. *Cell* 173 972–988. 10.1016/j.cell.2018.03.050 29656893 PMC8108093

[B50] Hammet A. Magill C. Heierhorst J. Jackson S. P. (2007). Rad9 BRCT domain interaction with phosphorylated H2AX regulates the G1 checkpoint in budding yeast. *EMBO Rep.* 8 851–857. 10.1038/sj.embor.7401036 17721446 PMC1973948

[B51] Hargreaves D. C. Crabtree G. R. (2011). ATP-dependent chromatin remodeling: genetics, genomics and mechanisms. *Cell Res.* 21 396–420. 10.1038/cr.2011.32 21358755 PMC3110148

[B52] He S. Wu Z. Tian Y. Yu Z. Yu J. Wang X. (2020). Structure of nucleosome-bound human BAF complex. *Science* 367 875–881. 10.1126/science.aaz9761 32001526

[B53] Hsu P. L. Shi H. Leonen C. Kang J. Chatterjee C. Zheng N. (2019). Structural basis of H2B ubiquitination-dependent H3K4 methylation by COMPASS. *Mol. Cell* 76 712–723. 10.1016/j.molcel.2019.10.013 31733991 PMC6948180

[B54] Huen M. S. Huang J. Leung J. W. Sy S. M.-H. Leung K. M. Ching Y.-P. (2010). Regulation of chromatin architecture by the PWWP domain-containing DNA damage-responsive factor EXPAND1/MUM1. *Mol. Cell* 37 854–864. 10.1016/j.molcel.2009.12.040 20347427 PMC3695488

[B55] Iwabuchi K. Bartel P. L. Li B. Marraccino R. Fields S. (1994). Two cellular proteins that bind to wild-type but not mutant p53. *Proc. Natl. Acad. Sci. U.S.A.* 91 6098–6102. 10.1073/pnas.91.13.6098 8016121 PMC44145

[B56] Jahn L. J. Mason B. Brøgger P. Toteva T. Nielsen D. K. Thon G. (2018). Dependency of heterochromatin domains on replication factors. *G3* 8 477–489. 10.1534/g3.117.300341 29187422 PMC5919735

[B57] Jang S. Kang C. Yang H.-S. Jung T. Hebert H. Chung K. Y. (2019). Structural basis of recognition and destabilization of the histone H2B ubiquitinated nucleosome by the DOT1L histone H3 Lys79 methyltransferase. *Genes Dev.* 33 620–625. 10.1101/gad.323790.118 30923167 PMC6546062

[B58] Javaheri A. Wysocki R. Jobin-Robitaille O. Altaf M. Côté J. Kron S. J. (2006). Yeast G1 DNA damage checkpoint regulation by H2A phosphorylation is independent of chromatin remodeling. *Proc. Natl. Acad. Sci. U.S.A.* 103 13771–13776. 10.1073/pnas.0511192103 16940359 PMC1564209

[B59] Kalocsay M. Hiller N. J. Jentsch S. (2009). Chromosome-wide Rad51 spreading and SUMO-H2A.Z-dependent chromosome fixation in response to a persistent DNA double-strand break. *Mol. Cell* 33 335–343. 10.1016/j.molcel.2009.01.016 19217407

[B60] Kang R. S. Daniels C. M. Francis S. A. Shih S. C. Salerno W. J. Hicke L. (2003). Solution structure of a CUE-ubiquitin complex reveals a conserved mode of ubiquitin binding. *Cell* 113 621–630. 10.1016/S0092-8674(03)00362-3 12787503

[B61] Kornberg R. D. (1974). Chromatin structure: a repeating unit of histones and DNA. *Science* 184 868–871. 10.1126/science.184.4139.868 4825889

[B62] Kornberg R. D. Thomas J. O. (1974). Chromatin structure: oligomers of the histones. *Science* 184 865–868. 10.1126/science.184.4139.865 4825888

[B63] Lademann C. A. Renkawitz J. Pfander B. Jentsch S. (2017). The INO80 complex removes H2A.Z to promote presynaptic filament formation during homologous recombination. *Cell Rep.* 19 1294–1303. 10.1016/j.celrep.2017.04.051 28514650

[B64] Lazzaro F. Sapountzi V. Granata M. Pellicioli A. Vaze M. Haber J. E. (2008). Histone methyltransferase Dot1 and Rad9 inhibit single-stranded DNA accumulation at DSBs and uncapped telomeres. *EMBO J.* 27 1502–1512. 10.1038/emboj.2008.81 18418382 PMC2328446

[B65] Lee J. Choi E. S. Seo H. D. Kang K. Gilmore J. M. Florens L. (2017). Chromatin remodeller Fun30(Fft3) induces nucleosome disassembly to facilitate RNA polymerase II elongation. *Nat. Commun.* 8:14527. 10.1038/ncomms14527 28218250 PMC5321744

[B66] Li J. Li Y. He Q. Li Y. Li H. Liu L. (2014). One-pot native chemical ligation of peptide hydrazides enables total synthesis of modified histones. *Organ. Biomol. Chem.* 12 5435–5441. 10.1039/C4OB00715H 24934931

[B67] Li J.-B. Qi Y.-K. He Q.-Q. Ai H.-S. Liu S.-L. Wang J.-X. (2018). Chemically synthesized histone H2A Lys13 di-ubiquitination promotes binding of 53BP1 to nucleosomes. *Cell Res.* 28 257–260. 10.1038/cr.2017.157 29243734 PMC5799816

[B68] Li M. Xia X. Tian Y. Jia Q. Liu X. Lu Y. (2019). Mechanism of DNA translocation underlying chromatin remodelling by Snf2. *Nature* 567 409–413. 10.1038/s41586-019-1029-2 30867599

[B69] Lim M. Newman J. A. Williams H. L. Masino L. Aitkenhead H. Gravard A. E. (2019). A ubiquitin-binding domain that binds a structural fold distinct from that of ubiquitin. *Structure* 27 1316–1325.e6. 10.1016/j.str.2019.05.003 31204252 PMC6688830

[B70] Liu X. Li M. Xia X. Li X. Chen Z. (2017). Mechanism of chromatin remodelling revealed by the Snf2-nucleosome structure. *Nature* 544 440–445. 10.1038/nature22036 28424519

[B71] Marks K. C. Banks W. R. III Cunningham D. Witman P. M. Herman G. E. (2014). Analysis of two candidate genes for Basan syndrome. *Am. J. Med. Genet. A* 164A 1188–1191. 10.1002/ajmg.a.36438 24664640

[B72] Matsuoka S. Ballif B. A. Smogorzewska A. McDonald E. R. Hurov K. E. Luo J. (2007). ATM and ATR substrate analysis reveals extensive protein networks responsive to DNA damage. *Science* 316 1160–1166. 10.1126/science.1140321 17525332

[B73] McGinty R. K. Kim J. Chatterjee C. Roeder R. G. Muir T. W. (2008). Chemically ubiquitylated histone H2B stimulates hDot1L-mediated intranucleosomal methylation. *Nature* 453 812–816. 10.1038/nature06906 18449190 PMC3774535

[B74] Mirman Z. Lottersberger F. Takai H. Kibe T. Gong Y. Takai K. (2018). 53BP1–RIF1–shieldin counteracts DSB resection through CST-and Polα-dependent fill-in. *Nature* 560 112–116. 10.1038/s41586-018-0324-7 30022158 PMC6072559

[B75] Morgan M. T. Haj-Yahya M. Ringel A. E. Bandi P. Brik A. Wolberger C. (2016). Structural basis for histone H2B deubiquitination by the SAGA DUB module. *Science* 351 725–728. 10.1126/science.aac5681 26912860 PMC4863942

[B76] Neves-Costa A. Will W. R. Vetter A. T. Miller J. R. Varga-Weisz P. (2009). The SNF2-family member Fun30 promotes gene silencing in heterochromatic loci. *PLoS One* 4:e8111. 10.1371/journal.pone.0008111 19956593 PMC2780329

[B77] Noordermeer S. M. Adam S. Setiaputra D. Barazas M. Pettitt S. J. Ling A. K. (2018). The shieldin complex mediates 53BP1-dependent DNA repair. *Nature* 560 117–121. 10.1038/s41586-018-0340-7 30022168 PMC6141009

[B78] Nousbeck J. Burger B. Fuchs-Telem D. Pavlovsky M. Fenig S. Sarig O. (2011). A mutation in a skin-specific isoform of SMARCAD1 causes autosomal-dominant adermatoglyphia. *Am. J. Hum. Genet.* 89 302–307. 10.1016/j.ajhg.2011.07.004 21820097 PMC3155166

[B79] Ouspenski I. I. Elledge S. J. Brinkley B. (1999). New yeast genes important for chromosome integrity and segregation identified by dosage effects on genome stability. *Nucleic Acids Res.* 27 3001–3008. 10.1093/nar/27.15.3001 10454593 PMC148523

[B80] Pan M. Zheng Q. Gao S. Qu Q. Yu Y. Wu M. (2019). Chemical synthesis of structurally defined phosphorylated ubiquitins suggests impaired parkin activation by phosphorylated ubiquitins with a non-phosphorylated distal unit. *CCS Chem.* 1 476–489. 10.31635/ccschem.019.20190001

[B81] Panier S. Boulton S. J. (2014). Double-strand break repair: 53BP1 comes into focus. *Nat. Rev. Mol. Cell Biol.* 15 7–18. 10.1038/nrm3719 24326623

[B82] Park S. H. Ayoub A. Lee Y.-T. Xu J. Kim H. Zheng W. (2019). Cryo-EM structure of the human MLL1 core complex bound to the nucleosome. *Nat. Communicat.* 10 1–13. 10.1038/s41467-019-13550-2 31804488 PMC6895043

[B83] Pfander B. Bantele S. (2019). Nucleosome remodeling by Fun30SMARCAD1 in the DNA damage response. *Front. Mol. Biosci.* 6:78. 10.3389/fmolb.2019.00078 31555662 PMC6737033

[B84] Pfander B. Diffley J. F. (2011). Dpb11 coordinates Mec1 kinase activation with cell cycle-regulated Rad9 recruitment. *EMBO J.* 30 4897–4907. 10.1038/emboj.2011.345 21946560 PMC3243626

[B85] Rowbotham S. P. Barki L. Neves-Costa A. Santos F. Dean W. Hawkes N. (2011). Maintenance of silent chromatin through replication requires SWI/SNF-like chromatin remodeler SMARCAD1. *Mol. Cell* 42 285–296. 10.1016/j.molcel.2011.02.036 21549307

[B86] Sachs P. Ding D. Bergmaier P. Lamp B. Schlagheck C. Finkernagel F. (2019). SMARCAD1 ATPase activity is required to silence endogenous retroviruses in embryonic stem cells. *Nat. Commun.* 10:1335. 10.1038/s41467-019-09078-0 30902974 PMC6430823

[B87] Schwartz M. F. Duong J. K. Sun Z. Morrow J. S. Pradhan D. Stern D. F. (2002). Rad9 phosphorylation sites couple Rad53 to the *Saccharomyces cerevisiae* DNA damage checkpoint. *Mol. Cell* 9 1055–1065. 10.1016/S1097-2765(02)00532-4 12049741

[B88] Setiaputra D. Durocher D. (2019). Shieldin–the protector of DNA ends. *EMBO Rep.* 20:e47560. 10.15252/embr.201847560 30948458 PMC6501030

[B89] Siman P. Karthikeyan S. V. Nikolov M. Fischle W. Brik A. (2013). Convergent chemical synthesis of histone H2B protein for the site-specific ubiquitination at Lys34. *Angew. Chem. Int. Ed.* 52 8059–8063. 10.1002/anie.201303844 23794525

[B90] Smolle M. Venkatesh S. Gogol M. M. Li H. Zhang Y. Florens L. (2012). Chromatin remodelers Isw1 and Chd1 maintain chromatin structure during transcription by preventing histone exchange. *Nat. Struct. Mol. Biol.* 19 884–892. 10.1038/nsmb.2312 22922743 PMC3560298

[B91] Steglich B. Stralfors A. Khorosjutina O. Persson J. Smialowska A. Javerzat J. P. (2015). The Fun30 chromatin remodeler Fft3 controls nuclear organization and chromatin structure of insulators and subtelomeres in fission yeast. *PLoS Genet.* 11:e1005101. 10.1371/journal.pgen.1005101 25798942 PMC4370569

[B92] Stralfors A. Walfridsson J. Bhuiyan H. Ekwall K. (2011). The FUN30 chromatin remodeler, Fft3, protects centromeric and subtelomeric domains from euchromatin formation. *PLoS Genet.* 7:e1001334. 10.1371/journal.pgen.1001334 21437270 PMC3060074

[B93] Sun Y. McCorvie T. J. Yates L. A. Zhang X. (2020). Structural basis of homologous recombination. *Cell Mol. Life Sci.* 77 3–18. 10.1007/s00018-019-03365-1 31748913 PMC6957567

[B94] Sweeney F. D. Yang F. Chi A. Shabanowitz J. Hunt D. F. Durocher D. (2005). *Saccharomyces cerevisiae* Rad9 acts as a Mec1 adaptor to allow Rad53 activation. *Curr. Biol.* 15 1364–1375. 10.1016/j.cub.2005.06.063 16085488

[B95] Taneja N. Zofall M. Balachandran V. Thillainadesan G. Sugiyama T. Wheeler D. (2017). SNF2 family protein Fft3 suppresses nucleosome turnover to promote epigenetic inheritance and proper replication. *Mol. Cell* 66 50–62.e6. 10.1016/j.molcel.2017.02.006 28318821 PMC5407362

[B96] Terui R. Nagao K. Kawasoe Y. Taki K. Higashi T. L. Tanaka S. (2018). Nucleosomes around a mismatched base pair are excluded via an Msh2-dependent reaction with the aid of SNF2 family ATPase Smarcad1. *Gen. Dev.* 32 806–821. 10.1101/gad.310995.117 29899141 PMC6049510

[B97] Toh G. W.-L. O’Shaughnessy A. M. Jimeno S. Dobbie I. M. Grenon M. Maffini S. (2006). Histone H2A phosphorylation and H3 methylation are required for a novel Rad9 DSB repair function following checkpoint activation. *DNA Rep.* 5 693–703. 10.1016/j.dnarep.2006.03.005 16650810

[B98] Ubersax J. A. Woodbury E. L. Quang P. N. Paraz M. Blethrow J. D. Shah K. (2003). Targets of the cyclin-dependent kinase Cdk1. *Nature* 425 859–864. 10.1038/nature02062 14574415

[B99] Uckelmann M. Densham R. M. Baas R. Winterwerp H. H. K. Fish A. Sixma T. K. (2018). USP48 restrains resection by site-specific cleavage of the BRCA1 ubiquitin mark from H2A. *Nat. Commun.* 9:229. 10.1038/s41467-017-02653-3 29335415 PMC5768779

[B100] Umebayashi K. (2003). The roles of ubiquitin and lipids in protein sorting along the endocytic pathway. *Cell Struct. Funct.* 28 443–453. 10.1247/csf.28.443 14745136

[B101] Valencia-Sanchez M. I. De Ioannes P. Wang M. Vasilyev N. Chen R. Nudler E. (2019). Structural basis of Dot1L stimulation by histone H2B lysine 120 ubiquitination. *Mol. Cell* 74 1010–1019.e6 10.1016/j.molcel.2019.03.029 30981630 PMC7009778

[B102] van Attikum H. Fritsch O. Gasser S. M. (2007). Distinct roles for SWR1 and INO80 chromatin remodeling complexes at chromosomal double-strand breaks. *EMBO J.* 26 4113–4125. 10.1038/sj.emboj.7601835 17762868 PMC2230671

[B103] VanDemark A. P. Kasten M. M. Ferris E. Heroux A. Hill C. P. Cairns B. R. (2007). Autoregulation of the rsc4 tandem bromodomain by gcn5 acetylation. *Mol. Cell* 27 817–828. 10.1016/j.molcel.2007.08.018 17803945 PMC2788556

[B104] Weinert T. A. Hartwell L. H. (1988). The RAD9 gene controls the cell cycle response to DNA damage in *Saccharomyces cerevisiae*. *Science* 241 317–322. 10.1126/science.3291120 3291120

[B105] WillhoftO.GhoneimM.LinC. L.ChuaE. Y. D.WilkinsonM.ChabanY. (2018). Structure and dynamics of the yeast SWR1-nucleosome complex. *Science* 362:eaat7716. 10.1126/science.aat7716 30309918

[B106] Wilson M. D. Benlekbir S. Fradet-Turcotte A. Sherker A. Julien J.-P. McEwan A. (2016). The structural basis of modified nucleosome recognition by 53BP1. *Nature* 536 100–103. 10.1038/nature18951 27462807

[B107] Worden E. J. Hoffmann N. A. Hicks C. W. Wolberger C. (2019). Mechanism of cross-talk between H2B ubiquitination and H3 methylation by Dot1L. *Cell* 176 1490.–1501. 10.1016/j.cell.2019.02.002 30765112 PMC6498860

[B108] Worden E. J. Wolberger C. (2019). Activation and regulation of H2B-Ubiquitin-dependent histone methyltransferases. *Curr. Opin. Struct. Biol.* 59 98–106. 10.1016/j.sbi.2019.05.009 31229920 PMC6888998

[B109] Wysocka J. Swigut T. Xiao H. Milne T. A. Kwon S. Y. Landry J. (2006). A PHD finger of NURF couples histone H3 lysine 4 trimethylation with chromatin remodelling. *Nature* 442 86–90. 10.1038/nature04815 16728976

[B110] Wysocki R. Javaheri A. Allard S. Sha F. Côté J. Kron S. J. (2005). Role of Dot1-dependent histone H3 methylation in G1 and S phase DNA damage checkpoint functions of Rad9. *Mol. Cell. Biol.* 25 8430–8443. 10.1128/MCB.25.19.8430-8443.2005 16166626 PMC1265753

[B111] Xiao S. Lu J. Sridhar B. Cao X. Yu P. Zhao T. (2017). SMARCAD1 contributes to the regulation of naive pluripotency by interacting with histone citrullination. *Cell Rep.* 18 3117–3128. 10.1016/j.celrep.2017.02.070 28355564 PMC5466819

[B112] Xu G. Chapman J. R. Brandsma I. Yuan J. Mistrik M. Bouwman P. (2015). REV7 counteracts DNA double-strand break resection and affects PARP inhibition. *Nature* 521 541–544. 10.1038/nature14328 25799992 PMC4671316

[B113] Xu Y. Ayrapetov M. K. Xu C. Gursoy-Yuzugullu O. Hu Y. Price B. D. (2012). Histone H2A.Z controls a critical chromatin remodeling step required for DNA double-strand break repair. *Mol. Cell* 48 723–733. 10.1016/j.molcel.2012.09.026 23122415 PMC3525728

[B114] Xue H. Yao T. Cao M. Zhu G. Li Y. Yuan G. (2019). Structural basis of nucleosome recognition and modification by MLL methyltransferases. *Nature* 573 445–449. 10.1038/s41586-019-1528-1 31485071

[B115] Yan L. Wu H. Li X. Gao N. Chen Z. (2019). Structures of the ISWI–nucleosome complex reveal a conserved mechanism of chromatin remodeling. *Nat. Struct. Mol. Biol.* 26 258–266. 10.1038/s41594-019-0199-9 30872815

[B116] Yao T. Jing W. Hu Z. Tan M. Cao M. Wang Q. (2019). Structural basis of the crosstalk between histone H2B monoubiquitination and H3 lysine 79 methylation on nucleosome. *Cell Res.* 29 330–333. 10.1038/s41422-019-0146-7 30770869 PMC6461977

[B117] Ye Y. Wu H. Chen K. Clapier C. R. Verma N. Zhang W. (2019). Structure of the RSC complex bound to the nucleosome. *Science* 366 838–843. 10.1126/science.aay0033 31672915 PMC8442553

[B118] Yu Q. Zhang X. Bi X. (2011). Roles of chromatin remodeling factors in the formation and maintenance of heterochromatin structure. *J. Biol. Chem.* 286 14659–14669. 10.1074/jbc.M110.183269 21388962 PMC3077663

[B119] Zhou C. Y. Johnson S. L. Gamarra N. I. Narlikar G. J. (2016). Mechanisms of ATP-dependent chromatin remodeling motors. *Annu. Rev. Biophys.* 45 153–181. 10.1146/annurev-biophys-051013-022819 27391925 PMC9157391

[B120] Zhou L. Holt M. T. Ohashi N. Zhao A. Muller M. M. Wang B. (2016). Evidence that ubiquitylated H2B corrals hDot1L on the nucleosomal surface to induce H3K79 methylation. *Nat. Commun.* 7:10589. 10.1038/ncomms10589 26830124 PMC4740876

